# Self-Administered Interventions Based on Natural Language Processing Models for Reducing Depressive and Anxiety Symptoms: Systematic Review and Meta-Analysis

**DOI:** 10.2196/59560

**Published:** 2024-08-21

**Authors:** David Villarreal-Zegarra, C Mahony Reategui-Rivera, Jackeline García-Serna, Gleni Quispe-Callo, Gabriel Lázaro-Cruz, Gianfranco Centeno-Terrazas, Ricardo Galvez-Arevalo, Stefan Escobar-Agreda, Alejandro Dominguez-Rodriguez, Joseph Finkelstein

**Affiliations:** 1 Instituto Peruano de Orientación Psicológica Lima Peru; 2 Department of Biomedical Informatics School of Medicine University of Utah Salt Lake City, UT United States; 3 Instituto Nacional de Salud del Niño San Borja Lima Peru; 4 Telehealth Unit Universidad Nacional Mayor de San Marcos Lima Peru; 5 Department of Psychology, Health, and Technology University of Twente Enschede Netherlands

**Keywords:** natural language processing, depression, anxiety, systematic review, artificial intelligence, AI

## Abstract

**Background:**

The introduction of natural language processing (NLP) technologies has significantly enhanced the potential of self-administered interventions for treating anxiety and depression by improving human-computer interactions. Although these advances, particularly in complex models such as generative artificial intelligence (AI), are highly promising, robust evidence validating the effectiveness of the interventions remains sparse.

**Objective:**

The aim of this study was to determine whether self-administered interventions based on NLP models can reduce depressive and anxiety symptoms.

**Methods:**

We conducted a systematic review and meta-analysis. We searched Web of Science, Scopus, MEDLINE, PsycINFO, IEEE Xplore, Embase, and Cochrane Library from inception to November 3, 2023. We included studies with participants of any age diagnosed with depression or anxiety through professional consultation or validated psychometric instruments. Interventions had to be self-administered and based on NLP models, with passive or active comparators. Outcomes measured included depressive and anxiety symptom scores. We included randomized controlled trials and quasi-experimental studies but excluded narrative, systematic, and scoping reviews. Data extraction was performed independently by pairs of authors using a predefined form. Meta-analysis was conducted using standardized mean differences (SMDs) and random effects models to account for heterogeneity.

**Results:**

In all, 21 articles were selected for review, of which 76% (16/21) were included in the meta-analysis for each outcome. Most of the studies (16/21, 76%) were recent (2020-2023), with interventions being mostly AI-based NLP models (11/21, 52%); most (19/21, 90%) delivered some form of therapy (primarily cognitive behavioral therapy: 16/19, 84%). The overall meta-analysis showed that self-administered interventions based on NLP models were significantly more effective in reducing both depressive (SMD 0.819, 95% CI 0.389-1.250; *P*<.001) and anxiety (SMD 0.272, 95% CI 0.116-0.428; *P*=.001) symptoms compared to various control conditions. Subgroup analysis indicated that AI-based NLP models were effective in reducing depressive symptoms (SMD 0.821, 95% CI 0.207-1.436; *P*<.001) compared to pooled control conditions. Rule-based NLP models showed effectiveness in reducing both depressive (SMD 0.854, 95% CI 0.172-1.537; *P*=.01) and anxiety (SMD 0.347, 95% CI 0.116-0.578; *P*=.003) symptoms. The meta-regression showed no significant association between participants’ mean age and treatment outcomes (all *P*>.05). Although the findings were positive, the overall certainty of evidence was very low, mainly due to a high risk of bias, heterogeneity, and potential publication bias.

**Conclusions:**

Our findings support the effectiveness of self-administered NLP-based interventions in alleviating depressive and anxiety symptoms, highlighting their potential to increase accessibility to, and reduce costs in, mental health care. Although the results were encouraging, the certainty of evidence was low, underscoring the need for further high-quality randomized controlled trials and studies examining implementation and usability. These interventions could become valuable components of public health strategies to address mental health issues.

**Trial Registration:**

PROSPERO International Prospective Register of Systematic Reviews CRD42023472120; https://www.crd.york.ac.uk/prospero/display_record.php?ID=CRD42023472120

## Introduction

### Background

Depression and anxiety pose a substantial worldwide burden. In 2020, depression and anxiety affected approximately 246 million and 374 million people, respectively [1]. Moreover, these conditions reduce individuals’ quality of life and have significant economic repercussions [2]. The World Health Organization estimates that depression and anxiety result in a loss of US $1 trillion annually due to loss of productivity [3]. In addition, their increasing incidence and a lack of health resources challenge the health care systems and workforce to meet the growing demand for mental health care services adequately [4].

In response, self-administered technology-based interventions have emerged as promising solutions for managing these conditions. These self-guided interventions enable users to progress through treatments independently, without external support [4], and they have demonstrated the potential to reduce costs; save health providers’ time; and improve satisfaction and access to care, especially during crises and quarantine periods, for patients with mental health conditions living in remote areas, those with disabilities, or those unable to afford traditional care [5]. However, despite the potential of self-directed interventions to manage mental health problems, many of these interventions face important challenges in user engagement and adherence [6].

Self-administered interventions that are effective vary by delivery format, including web-based platforms, mobile apps, and virtual or augmented reality [7,8]. These interventions can be integrated within a professional intervention package or be completely independent of any external support [9,10]. Furthermore, they can be based solely on the presentation of relevant therapeutic information, typically based on a behavioral cognitive approach [10-12], or rely on machine learning (ML) models to process the natural language of clients’ responses [13].

Natural language processing (NLP) offers a promising avenue for enhancing the efficacy of self-administered interventions. Defined as a cross-disciplinary field focused on enabling computers to comprehend, process, and interact with human language [14], NLP has the potential to make self-directed interventions more cost-effective and accessible and facilitate fidelity and engagement of patients through better interaction [15].

Moreover, NLP can be categorized into 2 main approaches: rule based and artificial intelligence (AI) based. Rule-based NLP uses predefined linguistic rules to guide text interpretation, offering high explainability but limited flexibility in handling complex language nuances [16]. Conversely, AI-based NLP, encompassing ML and deep learning techniques, learns from extensive data to process language. It has shown remarkable success in various NLP tasks due to its scalability and ability to manage linguistic ambiguities [17].

The advent of large language models and multimodal large language models has further enhanced the capabilities of NLP-based health interventions. These advances are not limited to enhanced user interaction but extend to personalizing therapeutic modalities to the patient’s unique requirements, as demonstrated in specific psychotherapeutic settings [18].

Previously, other systematic reviews, such as those conducted by Le Glaz et al [19] and Zhang et al [20], analyzed the impact of NLP on mental health. However, these reviews primarily focused on the general applications of NLP in mental health. In addition, another systematic review demonstrated promising results for NLP-based interventions in mental health, but the findings encompassed a broad range of mental health disorders and did not specifically address self-administered interventions [15].

### Objectives

Although these advances are highly promising, analysis of their effectiveness and safety in managing mental health concerns such as depression and anxiety remains fragmented [21]. This study aims to systematically review available literature to determine the effect of self-administered NLP-based interventions on symptoms of depression and anxiety.

## Methods

### Design and Protocol Registration

This study systematically searched available literature in the principal health databases and synthesized the main quantitative results in a meta-analysis. Our study adheres to the PRISMA (Preferred Reporting Items for Systematic Reviews and Meta-Analyses) guidelines (refer to Multimedia Appendix 1 for the PRISMA 2020 checklist) and the Cochrane Collaboration recommendations for meta-analyses [22]. The protocol for this systematic review was registered in the PROSPERO repository (CRD42023472120).

### Eligibility Criteria

Our study follows the PICOS (Population, Intervention, Comparison, Outcomes, and Study Design) framework to evaluate whether interventions based on NLP models can effectively reduce depressive and anxiety symptoms. We define these symptoms as follows: (1) depressive symptoms are defined as a mood disorder characterized by the persistent presence of a profound sense of sadness, loss of interest or pleasure in daily activities, and a general lack of energy; and (2) anxiety symptoms are characterized by the anticipation of imagined events that are perceived as potential threats, causing emotional distress and physiological tension.

The eligibility criteria for our review are presented in Textbox 1.

**Eligibility criteria for the review determined using the PICOS (Population, Intervention, Comparison, Outcomes, and Study Design) framework.**

**Review eligibility criteria**

- Population: we included studies with participants of any age group (child, adolescent, adult, and older adult) with or without previous comorbidities. Eligible studies must report participants who have been diagnosed with depression or anxiety through an interview or consultation with a mental health professional (eg, physician, psychologist, or psychiatrist) or assessed using validated psychometric instruments.
- Intervention: the intervention must be based on natural language processing (NLP) models such as large language models, multimodal large language models, artificial intelligence–led systems (ie, digital conversational agent, chatbot, or interactive voice response), and other NLP models. We included interventions regardless of their primary design purpose, provided they were self-administered.
- Comparison: we considered both passive (ie, waiting lists, nonintervention control groups, or placebos) and active (ie, web-based or face-to-face psychological interventions, virtual reality, serious games, biofeedback for mental health problems, pharmacological therapies to treat symptoms of depression and anxiety, or animal-assisted therapies) comparators.
- Outcomes: we included studies measuring depressive and anxiety symptom scores using validated psychometric questionnaires (eg, Patient Health Questionnaire-9, Beck Depression Inventory, Hamilton Depression Rating Scale, Generalized Anxiety Disorder-7, Beck Anxiety Inventory, Hamilton Anxiety Rating Scale, or similar instruments).
- Study Design: we included randomized controlled trials and quasi-experimental studies (without a control arm or randomization groups) that assessed the effect of NLP-based interventions on depressive and anxiety symptoms. We excluded narrative reviews, systematic reviews, scoping reviews, and other nonoriginal research designs. Only peer-reviewed publications (original articles or briefs) were included; proceedings, posters, and other similar items were excluded. There were no exclusion criteria based on language, publication date, or setting (ie, clinical or community settings).

### Information Sources and Search Strategy

The databases we used for the systematic review were Web of Science, Scopus, MEDLINE (by PubMed), PsycINFO (by EBSCO), IEEE Xplore, Embase, and Cochrane Library. The search strategy included terms related to NLP as well as depression and anxiety, along with health science descriptors (refer to Multimedia Appendix 2 for the search strategy). Our search included any document available from inception to November 3, 2023.

### Selection Process

We downloaded all records identified by the search strategy in RIS format and compiled them into an EndNote (Clarivate) file, which served as a repository for all retrieved records. Next, we used automated and manual methods to remove duplicate records. We exported the list of unique records from EndNote to Rayyan (Rayyan Systems Inc) for the selection process. First, 2 pairs of authors (JG-S with RG-A and GQ-C with GL-C) independently assessed the abstracts and titles of the studies to ensure that they met the inclusion criteria. Two pairs of authors reviewed the resulting retrieved text independently (JG-S with RG-A and GQ-C with GL-C). Any excluded studies were recorded along with the reasons for their exclusion (refer to Multimedia Appendix 3 for a list of the excluded studies). If disagreements arose between the reviewers at either stage, they were resolved by discussion. A third reviewer (DV-Z) was consulted if disagreement persisted to decide whether the study met the inclusion criteria. Records were included or excluded depending on whether they met the inclusion criteria. At the title and abstract stage, if it was unclear whether a record met all the inclusion criteria, it could proceed to the full-text stage, where a more detailed review was carried out (a sensitive approach). However, at the full-text stage, all inclusion criteria had to be met for final acceptance.

The title and abstract review were performed in English because this is the language in which the databases save the metadata. The full-text review and results extraction were mainly performed in English and Spanish (the languages the reviewers speak). When studies in other languages were found, the reviewers used DeepL Translator (DeepL SE) to translate the documents into English before proceeding with the review and extraction. Therefore, our review had no language limitations. It is important to note that all papers evaluated in the full-text review and extraction were in English.

### Data Collection

Two pairs of authors (JGS with RGA and GQC with GLC) independently collected the information from the included studies using a predefined collection form in a Microsoft Excel sheet. Initially, a pilot data extraction process was conducted on 5 data sets reviewed by all raters with 85% agreement. Subsequently, minor changes were made to the final version of the extraction form to improve the clarity of the extracted data, which included the following: (1) general information (ie, authors, year of publication, title, country, and language); (2) participant characteristics (ie, age range, sex, number of participants, and diagnosis); (3) intervention characteristics (ie, type of NLP model, duration, frequency, and brief description of the intervention); (4) comparator (passive or active); and (5) main outcomes (ie, means, SDs, preintervention and postintervention measures, and the effect size for control and intervention groups).

### Risk-of-Bias Assessment and Certainty of Evidence

We used the JBI critical appraisal tools to identify potential biases that may have occurred during the design, conduct, and analysis of the studies. For quasi-experimental studies, we used the JBI critical appraisal checklist for quasi-experimental studies [23], which is a checklist with 9 questions for assessing potential bias. For randomized controlled trials (RCTs), we used the JBI critical appraisal tool for the assessment of risk of bias in RCTs [24], which is a 13-question checklist evaluating the internal and statistical validity of the conclusions of RCTs. On the basis of the answers from both assessment tools, reviewers decide whether to include the reviewed study. Two reviewers used these tools independently to assess the risk of bias in the studies included in the meta-analysis. Any disagreement between the reviewers about whether to include or exclude a study was resolved by discussion. If the disagreement persisted, a third reviewer was asked to arbitrate.

We used the Grading of Recommendations Assessment, Development, and Evaluation (GRADE) methodology to assess the certainty of evidence regarding the intervention’s effects. This methodology evaluates the certainty of evidence based on several criteria, including risk of bias, inconsistency, indirectness, and imprecision [25]. Given that the GRADE approach is primarily focused on RCTs, and the GRADE working group has not yet reached a consensus on the combination of results from randomized and nonrandomized trials, we applied this evaluation exclusively to the RCTs included in our review.

### Synthesis Methods

#### Narrative Synthesis

To address the multifaceted nature of the factors involved in self-administered NLP-based interventions for symptoms of depression and anxiety, we adopted a comprehensive framework for data synthesis based on an adaptation of the categories from the framework for NLP applications for mental health interventions proposed by Malgaroli et al [15] in the context of self-administered NLP interventions. This systematic approach thoroughly integrates all relevant factors, providing a coherent structure for our analysis. We categorized data from eligible studies into four primary domains: (1) demographic and sample descriptions, (2) NLP technical aspects, (3) clinical categories, and (4) intervention results. Due to the nature of our study, the last category is presented through the findings of the meta-analysis and analysis of subgroups.

#### Meta-Analysis

We performed analyses using Stata (version 18.0; StataCorp LLC). Meta-analysis was only performed if at least 3 studies of the same design type (ie, randomized or quasi-experimental controlled trials) assessing the same outcome were available. The analysis was differentiated by outcome and by study type. Standardized mean differences (SMDs) with 95% CIs were used for meta-analyses and summary statistics of the studies because the results of the included studies were measured using different scales. SMD is the mean difference between the intervention and control groups divided by the pooled SD.

The standard measure of effect size to be considered for the Hedges *g* analyses includes small (SMD 0.2), moderate (SMD 0.5), and large (SMD >0.8) effect sizes. These thresholds were used to evaluate the combined effect of the analyzed interventions using Hedges *g*. Hedges *g*, unlike Cohen *d*, corrects for possible risk of bias associated with small sample sizes, making it a more appropriate measure for our analyses [26].

#### Heterogeneity Analysis

The assessment of statistical heterogeneity involved the following tests: the Cochran Q test statistic to detect the presence of heterogeneity between studies, the *I*² Higgins and *H*² index statistics to measure the extent of variability between studies due to heterogeneity, and the between-study variance (τ²) to assess the variance between the effects observed across the studies. If the overall assessment indicated high heterogeneity, random effects models were used to estimate the effect of the interventions in general.

#### Publication Bias Analysis

If there were >10 studies in the meta-analysis, we conducted both visual and quantitative tests to detect biases. Our visual examination used a funnel plot; the quantitative test used was the Egger regression test, which can capture the effects of small studies and other potential information biases [27]. We identified selection bias if we observed an asymmetric funnel plot distribution and a significant Egger regression test result (*P*<.05). In cases of asymmetry, the trim-and-fill method proposed by Duval and Tweedie [28] was implemented as a bias correction technique to estimate the number of missing studies for the meta-analysis.

#### Analysis of Subgroups

If the meta-analysis data allowed, we assessed intervention effects using the NLP-based models from the selected studies. Such models could include rule-based NLP, AI-based NLP, or other NLP. In addition, we assessed the impact of interventions on subgroups, including gender, disease severity, prior therapies, concurrent depression and anxiety disorders, and age ranges.

We performed a random effects meta-regression using aggregate-level data. Our analysis specified the variables containing the SE within each study using the *metareg* command and the *wsse* option in Stata. The meta-regression was a function of the mean age of the participants and was only applied to the overall meta-analysis. Our analysis yielded a meta-regression coefficient with 95% CI.

## Results

### Study Selection

**Figure 1:**
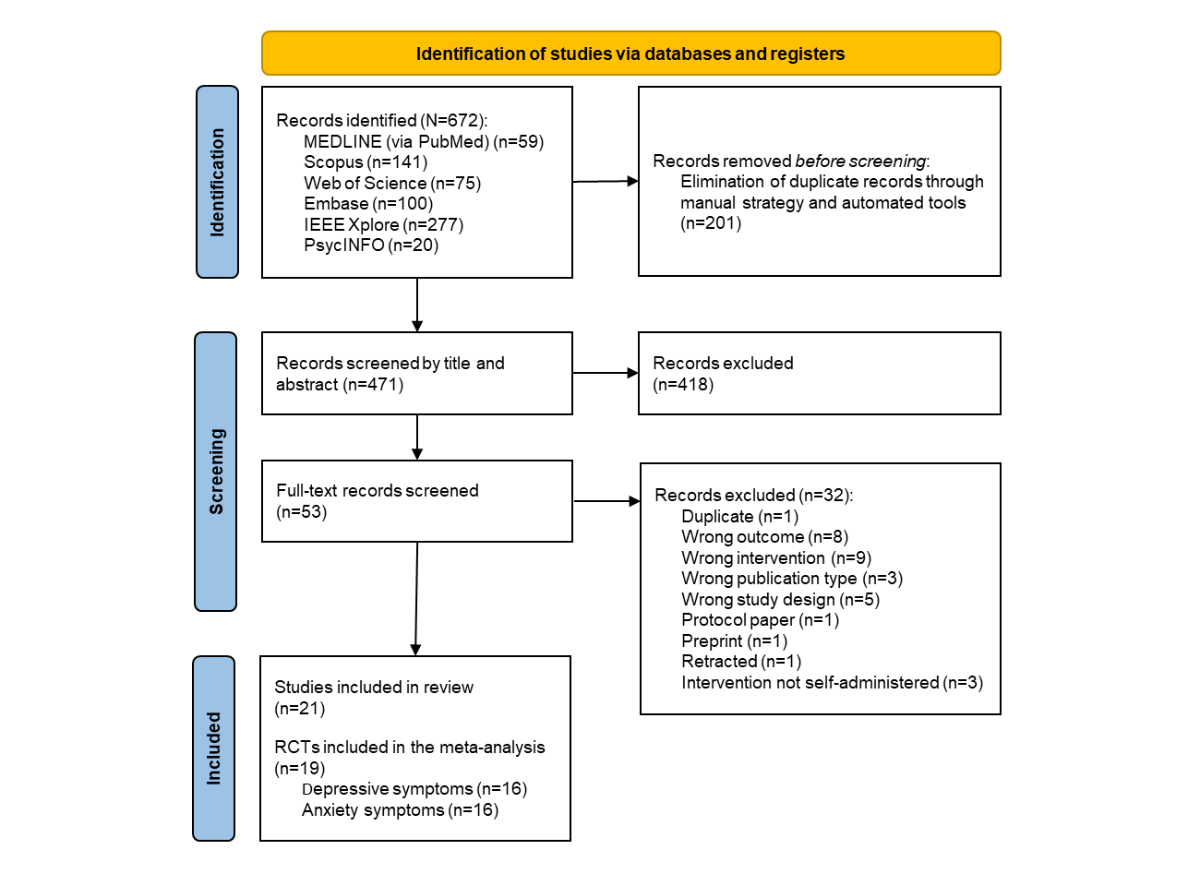
PRISMA (Preferred Reporting Items for Systematic Reviews and Meta-Analyses) flowchart of the selection process. RCT: randomized clinical trial.

Initially, 672 records were identified in the different databases; after eliminating 201 (29.9%) duplicates, 471 (70.1%) records advanced to title and abstract review. Of these 471 records, 418 (88.7%) were discarded, leaving 53 (11.3%) records for full-text review. Subsequently, 32 (60%) of the 53 records were excluded, resulting in 21 (40%) articles selected for review. Of these 21 articles, 19 (90%) were included in the meta-analysis on depressive and anxiety symptoms. Of the 19 studies included in the meta-analysis, 16 (84%) reported sufficient data for the meta-analysis of depressive symptoms, and another 16 (84%) reported sufficient data for the meta-analysis of anxiety symptoms. Figure 1 shows the complete review process, and Multimedia Appendices 3 and 4 [29-49] list the articles excluded and included, respectively.

### Characteristics of the Included Studies

Of the 21 studies identified, 19 (90%) were RCTs [29-47], and 2 (10%) were quasi-experimental studies without a control group [48,49]. Most of the studies (16/21, 76%) were published between 2020 and 2023, and 81% (17/21) were conducted in high-income countries. The United States was the country with the most publications among the selected studies (10/21, 48%). Regarding the characteristics of the populations studied, the majority (16/21, 76%) focused on adults. With regard to the outcomes assessed, depressive symptoms were analyzed in 95% (20/21) of the studies and anxiety symptoms in 90% (19/21). We found 29 potential comparisons between interventions and controls because 5 (24%) of the 21 studies reported ≥3 arms. AI-based NLP applications were the most common intervention (11/21, 52%), while the most common control conditions were waiting list or no intervention (8/21, 38%) and information, psychoeducation, or bibliotherapy (8/21, 38%). The most commonly used scales to measure depressive and anxiety symptoms were the Patient Health Questionnaire (PHQ; PHQ-9 and PHQ-8; 13/21, 62%) and the Generalized Anxiety Disorder-7 (GAD-7; 10/21, 48%), respectively. Table 1 shows the characteristics of the studies, divided into RCTs and uncontrolled quasi-experimental studies.

**Table 1:** Characteristics of the included studies (n=21).

Characteristics		Randomized controlled trials (n=19), n (%)	Uncontrolled quasi-experimental studies (n=2), n (%)
**Publication year**			
	2014-2015	1 (5)	0 (0)
	2016-2019	4 (21)	0 (0)
	2020-2023	14 (74)	2 (100)
**Country income level**			
	Upper-middle income	4 (21)	0 (0)
	High income	15 (79)	2 (100)
**Country**			
	Argentina	1 (5)	0 (0)
	China	3 (16)	0 (0)
	Italy	1 (5)	1 (50)
	Japan	1 (5)	0 (0)
	South Korea	2 (11)	0 (0)
	United Kingdom	2 (11)	0 (0)
	United States	9 (47)	1 (50)
**Study design**			
	Crossover	5 (26)	0 (0)
	Parallel	14 (74)	0 (0)
	Not applicable	0 (0)	2 (100)
**Participants’ life stage**			
	Adolescent	2 (11)	0 (0)
	Adult	14 (74)	2 (100)
	Older adult	2 (11)	0 (0)
	Pregnant	1 (5)	0 (0)
**Included in meta-analysis**			
	Depressive symptoms	16 (84)	0 (0)
	Anxiety symptoms	16 (84)	0 (0)
	Not applicable	0 (0)	2 (100)
**Depressive symptoms**			
	Main outcome	11 (58)	2 (100)
	Secondary outcome	7 (37)	0 (0)
	Not evaluated	1 (5)	0 (0)
**Anxiety symptoms**			
	Main outcome	11 (58)	2 (100)
	Secondary outcome	6 (32)	0 (0)
	Not evaluated	2 (11)	0 (0)
**Funding**			
	Corporations	8 (42)	1 (50)
	Government	4 (21)	0 (0)
	Self-financed	2 (11)	1 (50)
	Not reported	5 (26)	0 (0)
**Conflicts of interest**			
	Yes	4 (21)	2 (100)
	No	12 (63)	0 (0)
	Not reported	3 (16)	0 (0)
**Study has ≥3 arms**			
	No	14 (74)	2 (100)
	Yes	5 (26)	0 (0)
**Control group^a^**			
	Waiting list or no intervention	8 (42)	0 (0)
	Usual treatment	2 (11)	0 (0)
	Information, psychoeducation, or bibliotherapy	8 (42)	0 (0)
	Conversational computer-based intervention	5 (26)	0 (0)
	Not applicable	0 (0)	2 (100)
**Type of NLP^b^ application^a^**			
	Rule based	10 (53)	1 (50)
	AI^c^ based	11 (58)	1 (50)
**Focus of intervention^a^**			
	Depressive symptoms	8 (42)	1 (50)
	Anxiety symptoms	7 (37)	1 (50)
	Other mental health problems	13 (68)	2 (100)
**Therapeutical approach^a^**			
	Cognitive behavioral therapy	15 (79)	2 (100)
	Other	3 (16)	0 (0)
	Unclear	1 (5)	0 (0)
**Scale used to measure depression^a^**			
	PHQ^d^-9 and PHQ-8	13 (68)	2 (100)
	DASS-21^e^	2 (11)	0 (0)
	Other	3 (16)	0 (0)
	Not evaluated	1 (5)	0 (0)
**Scale used to measure anxiety^a^**			
	GAD-7^f^	10 (53)	2 (100)
	DASS-21	3 (16)	0 (0)
	Other	4 (21)	0 (0)
	Not evaluated	2 (11)	0 (0)

^a^The totals do not add up to 100% because there are studies with 3 and 4 arms that evaluated >1 type of intervention at the same time.

^b^NLP: natural language processing.

^c^AI: artificial intelligence.

^d^PHQ: Patient Health Questionnaire.

^e^DASS-21: Depression, Anxiety, and Stress Scale-21.

^f^GAD-7: Generalized Anxiety Disorder-7.

### NLP Technical Aspects

Of the 21 included studies, 10 (48) used rule-based approaches, while 11 (52%) used AI-based techniques. Within the AI-based category, of the 11 studies, 4 (36%) implemented deep learning methods, 6 (55%) did not specify the AI technique used, and 1 (9%) used ML algorithms. Regarding the specific NLP techniques used, sentiment analysis was used in 18% (2/11) of the studies, and natural language understanding was used in 18% (2/11). Notably, 7 (64%) of the 11 studies did not specify the NLP techniques used in their interventions. This distribution highlights a diverse application of NLP methods in addressing symptoms of depression and anxiety, with more than half of the studies (11/21, 52%) leveraging advanced AI techniques, albeit often without detailed specification (7/11, 64%).

The input modality for the NLP interventions was primarily text based in 19 (90%) of the 21 studies, while 1 (5%) study used either text or voice, and 1 (5%) study used voice alone. Regarding output modalities, text was predominantly used in 20 (95%) of the 21 studies, while only 1 (5%) study used voice. The language of the NLP input and output varied among the studies. Of the 21 studies, 7 (33%) used English, and 3 (14%) used Chinese, while Japanese, Spanish, and Italian were used in 1 (5%) study each. However, 38% (8/21) of the studies did not specify the language used for the NLP input and output.

### Demographics and Sample Descriptions

#### Overview

The study participants’ demographic characteristics were analyzed for rule-based NLP studies and AI-based NLP studies. All 21 studies provided demographic information regarding the sample or testing data set used for the intervention. Demographic data for rule-based NLP studies are reported only for the intervention samples. By contrast, AI-based NLP studies were expected to provide demographic information for the training data used to develop the AI-based models and the participants involved in the intervention or experiment.

#### Training Sample Description

None of the AI-based NLP studies provided detailed demographic information regarding the training data. While 3 (27%) of the 11 AI-based NLP studies mentioned the source of their training data (Stanford Sentiment Treebank data set, ad hoc user utterances from an unspecified source, and Emotion Support Conversation data set), they did not describe the demographic characteristics of these data sets.

#### Testing Data or Intervention Sample Description

Across all studies, gender distribution varied significantly. Of the 21 studies, in 3 (14%), only women participated; in 16 (76%), >50% of the participants were women; and in 2 (10%), >50% of the participants were men. Regarding the age of the participants, 20 (95%) of the 21 studies reported the mean age of their samples. Of these 20 studies, 9 (45%) involved participants aged >30 years, 10 (50%) included participants aged between 18 and 29 years, and 1 (5%) included participants aged <18 years. Participants’ special conditions were also considered in the analysis. Of the 21 studies, 4 (19%) included participants with chronic diseases, 7 (33%) focused on individuals with mental disorders, and 7 (33%) included university students, while 4 (19%) involved participants with other conditions. Specifically, among the 7 studies that focused on mental disorders, 2 (29%) included participants with a positive screening for depression, and 2 (29%) focused on participants with a positive screening for substance use disorder. Among the 4 studies that included participants with chronic diseases, there were diverse conditions, such as diabetes mellitus (n=1, 25%), cancer (n=1, 25%), inflammatory bowel disease (n=1, 25%), and dementia (n=1, 25%).

Focusing on the 11 AI-based NLP studies, the gender distribution of the intervention samples was as follows: in 9 (82%) studies, the majority of the participants were women; and in 2 (18%) studies, the majority of the participants were men. Regarding age distribution, of the 10 studies that reported mean ages, 5 (50%) involved participants aged >30 years, and 5 (50%) included participants aged between 18 and 29 years. With regard to special conditions in the intervention samples, of the 11 studies, 2 (18%) included participants with chronic diseases, 2 (18%) focused on individuals with mental disorders, 6 (55%) included university students, and 2 (18%) involved participants with other conditions (participants with panic disorder: n=1, 50%; and participants with a positive screening for depression: n=1, 50%). For chronic conditions, of the 2 studies, 1 (50%) involved patients with dementia, and 1 (50%) included patients with diabetes mellitus.

### Clinical Categories

The included studies were evaluated for their focus on clinical presentation and the delivery of therapeutic interventions. Only 1 (5%) of the 21 studies reported having a component of diagnosis and screening for mental health problems, although it did not specify the disease or the methods used for diagnosis.

Most of the studies (19/21, 90%) declared that they delivered some form of therapy through their NLP interventions. By contrast, 2 (10%) of the 21 studies did not include any therapeutic component. Among the 19 studies that delivered therapy, 16 (84%) implemented cognitive behavioral therapy, 1 (5%) combined cognitive behavioral therapy with dialectical behavioral therapy, and 2 (11%) reported delivering therapy but did not specify the therapeutic approach used.

### Meta-Analysis Findings

#### Main Meta-Analysis

**Figure 2:**
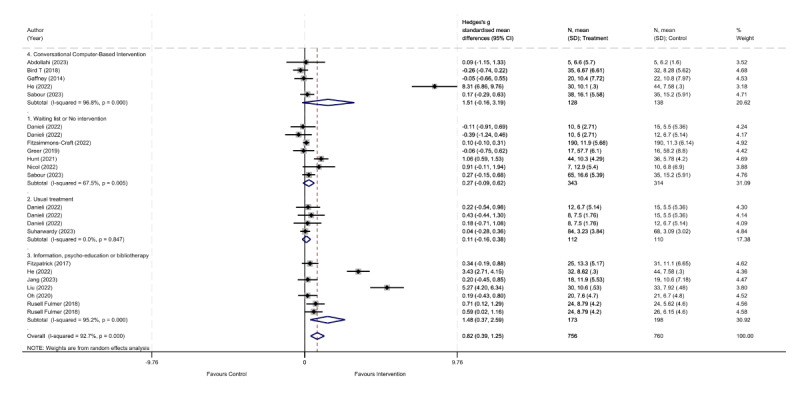
Forest plot for control conditions versus self-administered interventions based on natural language processing models to reduce depressive symptoms.

Only 16 (76%) of the 21 studies were included in the meta-analysis, excluding the uncontrolled quasi-experimental studies (n=2, 10%) and the RCTs with insufficient data for meta-analysis (n=3, 14%). The rationale for excluding the 2 quasi-experimental studies was that a meta-analysis specific to this study design required at least 3 studies of the same design type assessing the same outcome. For the depressive symptoms (Figure 2 [29-37,39-45]), the overall meta-analysis showed that self-administered interventions based on NLP models were significantly more effective in reducing depressive symptoms compared to various control conditions (waiting list or no intervention, treatment as usual, psychoeducation, and other computer-based conversational interventions; SMD 0.819, 95% CI 0.389-1.250; *P*<.001). In addition, high heterogeneity was observed in the overall meta-analysis (*I*^2^=92.7%, 95% CI 78.3%-96.4%; *H*^2^=3.71, 95% CI 2.15-5.27; τ^2^=0.97; *P*<.001). Regarding publication bias, the funnel plot analysis showed evidence of bias (Egger test coefficient=3.61, 95% CI 0.45-6.78; *P*=.03; Multimedia Appendix 5).

**Figure 3:**
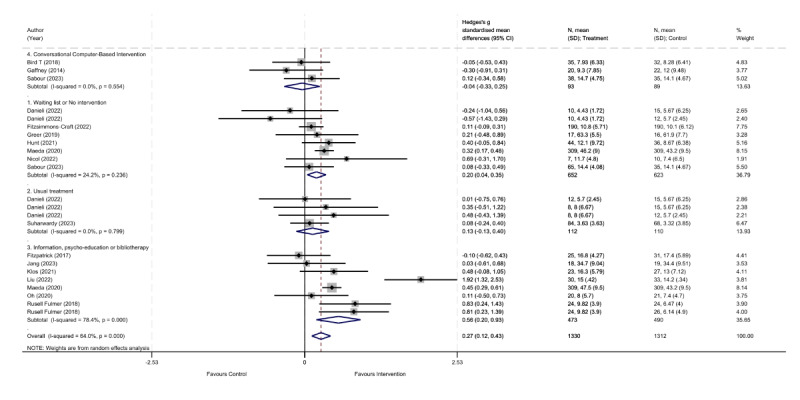
Forest plot for control conditions versus self-administered interventions based on natural language processing models to reduce anxiety symptoms.

For the outcome of anxiety symptoms (Figure 3 [30-34,36-46]), the global meta-analysis showed that self-administered NLP model–based interventions were significantly more effective in reducing depressive symptoms compared to various control conditions (waitlist or no intervention, treatment as usual, psychoeducation, and other conversational computer-based interventions; SMD 0.272; 95% CI 0.116-0.428; *P*=.001). In addition, high heterogeneity was observed in the overall meta-analysis (*I*^2^=64%, 95% CI 0.5%-81.6%; *H*^2^=1.67, 95% CI 1.00-2.33; τ^2^=0.07; *P*<.001). Regarding publication bias, the funnel plot analysis showed no evidence of bias (Egger test coefficient=–0.22, 95% CI –1.55 to 1.11; *P*=.73; Multimedia Appendix 5).

#### Subgroup Analyses

We also conducted a detailed analysis according to the type of comparator, intervention, and the scale used, evaluating the results for depressive symptoms and anxiety symptoms separately. For depressive symptoms, self-administered interventions based on NLP models were found to be more effective than information, psychoeducation, or bibliotherapy (SMD 1.481, 95% CI 0.368-2.594; *P*=.009). Similarly, AI-based NLP models were more effective than the set of control conditions (SMD 1.059, 95% CI 0.520-1.597; *P*<.001) for reducing depressive symptoms. Regarding the scale used, studies using the PHQ-9 or PHQ-8 showed that self-administered interventions based on NLP outperformed the set of control conditions (SMD 0.914, 95% CI 0.417-1.410; *P*<.001).

For the outcome of anxiety symptoms, self-administered interventions based on NLP models were more effective than waitlist or no intervention (SMD 0.196, 95% CI 0.042-0.351; *P*=.01) and information, psychoeducation, or bibliotherapy (SMD 0.561, 95% CI 0.195-0.927; *P*=.003). In addition, the use of AI-based NLP models had a higher effect than the average of the control conditions (SMD 0.302, 95% CI 0.073-0.532; *P*=.01) in reducing anxiety symptoms. Regarding the scale used, studies using the GAD-7 showed that self-administered interventions based on NLP had a higher effect than the average of the control conditions in reducing anxiety symptoms (SMD 0.333, 95% CI 0.074-0.592; *P*=.01). Full details of this subgroup analysis are presented in Table 2.

Given that factors such as age may influence the outcomes of depressive and anxiety symptoms, we performed a meta-regression to assess whether the mean age of participants affected the overall meta-analysis results. Our analysis revealed that the mean age was not significantly associated with the point estimates for either depressive symptoms (coefficient=–0.037, 95% CI –0.092 to 0.019; *P*=.18) or anxiety symptoms (coefficient=–0.010, 95% CI –0.030 to 0.010; *P*=.29). Detailed results of the meta-regression are presented in Table 3.

**Table 2:** Meta-analysis by subgroup for depressive and anxiety symptoms.

Symptoms and subgroups			Studies, n (%); groups, n	SMD^a^ (95% CI)	*P* value	Heterogeneity (*I*²; %)	Cochran Q test (*P* value)
**Depressive symptoms (n=16)**							
	**By control group**						
		Waiting list or no intervention	6 (38); 7	0.267 (–0.085 to 0.620)	.14	67.5	.005
		Usual treatment	2 (12); 4	0.111 (–0.155 to 0.378)	.41	0	.85
		Information, psychoeducation, or bibliotherapy	6 (38); 7	*1.481 (0.368 to 2.594)* ^b^	*.009*	95.2	<.001
		Conversational computer-based intervention	5 (31); 5	1.513 (–0.162 to 3.188)	.08	96.8	<.001
	**By intervention group**						
		Rule-based NLP^c^ model	7 (44); 7	*0.854 (0.172 to 1.537)*	*.01*	94	<.001
		AI^d^-based NLP model	9 (56); 16	*0.821 (0.207 to 1.436)*	*.009*	92.5	<.001
	**By scale used**						
		PHQ^e^-9 and PHQ-8	11 (69); 17	*0.914 (0.417 to 1.410)*	*<.001*	92.8	<.001
		DASS-21^f^	2 (12); 2	—^g^	—	—	—
**Anxiety symptoms (n=16)**							
	**By control group**						
		Waiting list or no intervention	7 (44); 8	*0.196 (0.042 to 0.351)*	*.01*	24.2	.24
		Usual treatment	2 (12); 4	0.133 (–0.134 to 0.400)	.33	0	.80
		Information, psychoeducation, or bibliotherapy	7 (44); 8	*0.561 (0.195 to 0.927)*	*.003*	78.4	<.001
		Conversational computer-based intervention	3 (19); 3	–0.041 (–0.333 to 0.250)	.78	0	.55
	**By intervention group**						
		Rule-based NLP model	8 (50); 9	*0.347 (0.116 to 0.578)*	*.003*	79.7	<.001
		AI-based NLP model	8 (50); 14	0.198 (–0.011 to 0.406)	.06	34.4	.10
	**By scale used**						
		GAD-7^h^	9 (56); 15	*0.333 (0.074 to 0.592)*	*.01*	71.7	<.001
		DASS-21	3 (19); 3	0.050 (–0.352 to 0.453)	.81	47.3	.15

^a^SMD: standardized mean difference.

^b^Italicized values are significant. Only meta-analyses with at least 3 measurements are presented in this study.

^c^NLP: natural language processing.

^d^AI: artificial intelligence.

^e^PHQ: Patient Health Questionnaire.

^f^DASS-21: Depression, Anxiety, and Stress Scale-21.

^g^There are not enough trials to do a meta-analysis.

^h^GAD-7: Generalized Anxiety Disorder-7.

**Table 3:** Meta-regression analysis by overall meta-analysis of depressive and anxiety symptoms.

Variable			Coefficient (SE; 95% CI)		*t (df)*		*P* value
**Depressive symptoms**							
	Age, mean	–0.037 (0.026; –0.092 to 0.019)		–1.390 (18)		.18	
	Intercept	2.108 (1.033; –0.063 to 4.279)		2.040 (18)		.06	
**Anxiety symptoms**							
	Age, mean	–0.010 (0.009; –0.030 to 0.010)		–1.080 (16)		.29	
	Intercept	0.553 (0.329; –0.145 to 1.251)		1.680 (16)		.11	

#### Risk of Bias and Certainty of Evidence

**Figure 4:**
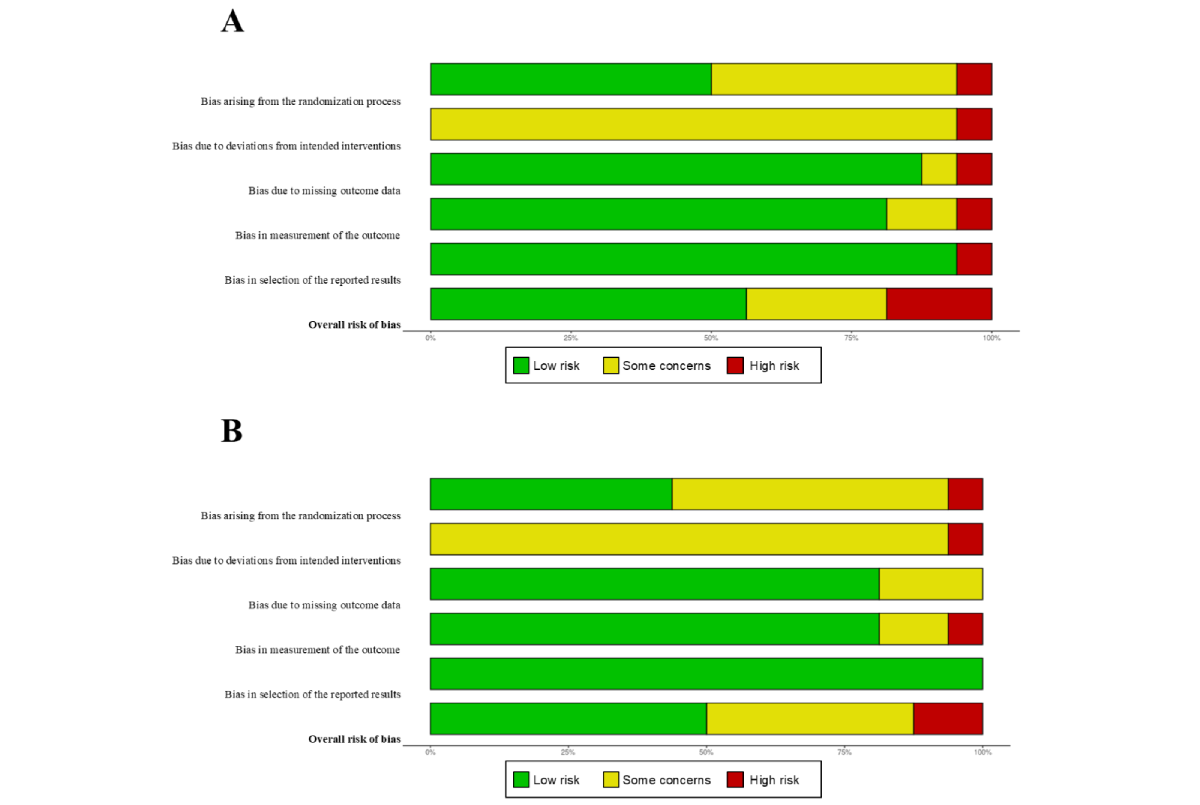
Risk of bias grouped for the outcomes of (A) depressive symptoms and (B) anxiety symptoms.

In the overall analysis of the risk of bias for the outcome of depressive symptoms, the majority of the studies (9/16, 56%) had an overall low risk of bias, while only 19% (3/16) had an overall high risk of bias (Figure 4A). Regarding the dimensions assessed, the lowest risk of bias was observed in reporting and analysis strategies (15/16, 94%), followed by participant loss or missing data (14/16, 88%). However, intervention delivery showed an unclear risk of bias due to limited reporting in the reviewed manuscripts. By contrast, for the outcome of anxiety symptoms, half of the studies (8/16, 50%) had an overall low risk of bias, while only 12% (2/16) had an overall high risk of bias (Figure 4B). At the level of each dimension assessed, all studies had a low risk of bias in reporting and analysis strategies, and 81% (13/16) had a low risk of bias in outcome measurement and retention throughout the study. Detailed risk-of-bias analyses for each study are available in Multimedia Appendix 6 for depressive symptoms and Multimedia Appendix 7 for anxiety symptoms.

We found that, for the outcomes studied (depressive symptoms and anxiety symptoms), the evidence was of very low certainty (Table 4). This was mainly due to several factors. First, there was a high risk of bias, with 3 (19%) of the 16 studies presenting an overall high risk of bias for depressive symptoms and 2 (12%) of the 16 studies presenting an overall high risk of bias for anxiety symptoms. Second, there was significant inconsistency, as indicated by an overall *I*² value of >60%. In addition, indirectness was a major concern due to the high variability in the interventions, controls, and sample characteristics across the studies. Finally, publication bias was strongly suspected due to the marked right-side asymmetry revealed by the funnel plot. Notwithstanding these limitations, the findings provide a preliminary understanding of the potential effects of self-administered NLP-based interventions on depressive and anxiety symptoms.

**Table 4:** Summary of findings and certainty of evidence using the Grading of Recommendations Assessment, Development, and Evaluation methodology.

Outcome	Assessment of certainty of evidence							Effect: Hedges *g*, SMD^a^ (95% CI)		Certainty of evidence
	Studies (RCTs^b^), n (participants, n)	Risk of bias	Inconsistency	Indirectness	Imprecision	Publication bias				
Depressive symptoms	16 (1516; control: 760, intervention: 756)	Very serious^c^	Very serious^d^	Very serious^e^	Not serious	Strongly suspected^f^	0.82, lower (0.39-1.25)		⊕ΟΟΟ^g^	
Anxiety symptoms	16 (2642; control: 1312, intervention: 1330)	Very serious^h^	Very serious^d^	Very serious^e^	Not serious	Strongly suspected^f^	0.27, lower (0.12-0.43)		⊕ΟΟΟ	

^a^SMD: standardized mean difference.

^b^RCT: randomized controlled trial.

^c^Of the 16 studies, 3 (19%) present an overall high risk of bias.

^d^Overall *I*^2^ value >60%.

^e^There is a high variability in the interventions, controls, and sample characteristics.

^f^The funnel plot reveals a marked right-side asymmetry.

^g^Very low (each filled circle [⊕] signifies a higher level of certainty, while each empty circle [Ο] indicates a lower level of certainty).

^h^Of the 16 studies, 2 (12%) present an overall high risk of bias.

## Discussion

### Principal Findings

Our results indicate that self-administered interventions based on NLP models have a significant overall effect on reducing depressive and anxiety symptoms compared to various control conditions. Our study used random effects models to estimate this overall effect, thus accounting for heterogeneity among the interventions analyzed. Therefore, we consider the results to be robust. At the level of each intervention group and control group, we observed variability in their effectiveness in reducing symptoms of depression and anxiety, which could be due to the limited number of studies available for meta-analysis. In particular, conversational computer-based interventions were shown to be effective in reducing depressive and anxiety symptoms compared to pooled control conditions. In addition, NLP-based interventions overall outperformed psychoeducation and bibliotherapy in reducing both depressive and anxiety symptoms. Furthermore, these interventions were more effective than waitlist or no intervention in reducing anxiety symptoms.

These findings support the usefulness of self-administered NLP-based interventions in alleviating such common mental health problems as depressive and anxiety symptoms. Thus, they have the potential to be implemented in primary care settings, where they could represent a valuable public health strategy to improve the mental health of the population.

### Comparison With Other Studies

Our findings are consistent with previous research that has examined the application of NLP-based models at various stages of mental health care in both clinical and community settings [50-52], indicating that NLP-based interventions may effectively alleviate symptoms of emotional disorders. The robustness of our research is strengthened by the fact that most of the studies included in the meta-analysis of depressive (9/16, 56%) and anxiety symptoms (8/16, 50%) have a low risk of bias, indicating that our findings are derived from rigorous and reliable research.

A previous scoping review highlighted the heterogeneity of the tools used to assess the effects of dialogue interventions on mental health [53]. However, our review found that in the case of RCTs focusing on depressive and anxiety symptoms, validated instruments such as the PHQ-9 and GAD-7 were used, reducing the risk of bias and making the results more robust. Nevertheless, we highlight the lack of studies using experiential sampling or real-time measures to assess depressive and anxiety symptoms, which could provide a more accurate assessment of the impact of these self-administered NLP-based interventions.

The subgroup analysis showed variability in the effectiveness of the interventions in reducing depressive and anxiety symptoms, which may be due to the limited number of studies analyzed. Another possible explanation lies in the variety of NLP-based models used and their level of sophistication. Interventions using conversational agents based on advanced deep ML models showed significant results compared to other strategies, such as rule-based chatbots [54]. Unlike simpler NLP-based models, conversational agents offer better performance on various tasks [54]. However, more complex models also require high computational costs and large amounts of data for optimization [55,56], which may limit their adaptability to the different linguistic and cultural needs of different regions [57]. It is important to note that high-income countries have led research in this field and have advanced technological resources for developing these AI-based models compared to low- and middle-income countries [58,59]. This situation represents a challenge and a potential source of inequity in access to, and the implementation of, NLP-based interventions within public health systems.

### Implications for Clinical Practice and Public Health

A previous systematic review on the general use of NLP and ML in mental health also identified the potential of NLP-based interventions to improve population mental health [19]. However, our study differs in that it focuses only on self-applied interventions to reduce depressive and anxiety symptoms, thus contributing to a specific aspect of NLP-based interventions. Our study provides a valuable starting point for future research to confirm the effectiveness of NLP-based interventions in the real world and their ability to be implemented within the public health system. There is a need to evaluate the implementation and promotion of these interventions as part of mental health strategies because this could be an effective strategy to reduce depressive and anxiety symptoms in health service users [60,61]. Given their accessibility through digital platforms, these interventions have the potential to reduce the burden of depressive and anxiety disorders at the population level [62,63] while also being cost-effective and a way to optimize mental health resources [64]. To ensure successful implementation within the public health system, using the Artificial Intelligence–Quality Implementation Framework could be beneficial [65]. However, it is crucial to develop protocols that ensure confidentiality and respect for the ethics and privacy of patient data at all stages of implementation and use [66]. In addition, it is important to consider the digital determinants of health [67], such as access to appropriate devices, the internet, and stable connectivity, because these factors pose challenges for implementation in low- and middle-income countries.

### Strengths and Limitations

The main strength of our study is that we conducted an exhaustive review of available literature on the subject and that the main meta-analysis was based on RCTs, which is the most robust design for determining the effect of an intervention. However, our study has several limitations. First, the methodological variability of the included studies led to high heterogeneity in both outcomes, which could affect the interpretation of our findings despite using random effects models for their management. Second, the various measurement tools used in the studies could introduce measurement bias. However, we believe that our study minimized this risk by including only studies that used validated instruments and an effect size that controls for heterogeneity among measures such as the SMD. Third, the lack of clarity in the description of the studied groups may have introduced a risk of bias in assessing their effectiveness because there is no clear taxonomy for grouping NLP-based interventions. Fourth, the global meta-analysis for depressive symptoms identified the potential existence of publication bias, which could overestimate results in favor of trials with positive effects. Therefore, we encourage researchers to report their studies, even if they have negative results, to understand the effect of these interventions better. Fifth, variability in the standards for diagnosing and treating depression and anxiety, as well as in the criteria for determining recovery among the included studies, may have affected the interpretation of the efficacy of the interventions and the generalizability of the findings to different populations. This heterogeneity highlights the importance of considering the context in which NLP-based interventions are applied and the need to adapt them to the characteristics of different populations [11]. Finally, the GRADE assessment shows that the evidence for self-administered NLP-based interventions on depressive and anxiety symptoms is of very low certainty. This suggests caution in interpreting these potential benefits. High risk of bias, significant inconsistency (high *I*² values), and high indirectness complicate the findings. Suspected publication bias further skews the results because studies with nonsignificant or negative outcomes may be underreported. To overcome these limitations in future reviews, we recommend focusing on specific interventions and encouraging researchers to share their primary data to strengthen the quality and reliability of meta-analytic analyses.

### Conclusions

Our systematic review and meta-analysis support the use of self-administered interventions based on NLP models to reduce depressive and anxiety symptoms. These findings enhance the theoretical understanding of how advanced NLP tools can effectively deliver psychological therapy, improving cognitive and emotional self-regulation in individuals. By demonstrating the efficacy of various NLP-based interventions, our study advances the theoretical framework by elucidating the mechanisms through which these technologies can replicate and potentially enhance traditional therapeutic processes.

The integration of NLP with different therapeutic modalities offers a novel approach to mental health treatment, expanding the accessibility and scalability of evidence-based interventions. However, the certainty of evidence for the effectiveness of these interventions remains very low, primarily due to a high risk of bias, significant inconsistency, and indirectness in the included studies. Therefore, there is a crucial need for RCTs with larger sample sizes and rigorous methodologies to strengthen the inferential power of future meta-analyses.

Moreover, while our findings are encouraging, there is a need for systematic reviews that examine the implementation processes of these interventions in depth, as well as qualitative studies that evaluate their usability and feasibility. Such research will be essential for effectively recommending the adoption of NLP-based self-administered interventions in public health systems.

Our study provides a valuable starting point for future research to validate the efficacy and practical implementation of these interventions as components of standard mental health care. Ensuring their integration into public health strategies could enhance the mental health outcomes of diverse populations, particularly those who may have limited access to traditional therapeutic resources.

## Appendix

Multimedia Appendix 1 PRISMA (Preferred Reporting Items for Systematic Reviews and Meta-Analyses) 2020 checklist.

Multimedia Appendix 2 Search strategy.

Multimedia Appendix 3 Excluded records.

Multimedia Appendix 4 Included records.

Multimedia Appendix 5 Funnel plot by depressive and anxiety symptoms.

Multimedia Appendix 6 Risk of bias for individual studies for the outcome of depressive symptoms.

Multimedia Appendix 7 Risk of bias for individual studies for the outcome of anxiety symptoms.

## Abbreviations

AI: artificial intelligence
GAD-7: Generalized Anxiety Disorder-7
GRADE: Grading of Recommendations Assessment, Development, and Evaluation
ML: machine learning
NLP: natural language processing
PHQ: Patient Health Questionnaire
PICOS: Population, Intervention, Comparison, Outcomes, and Study Design
PRISMA: Preferred Reporting Items for Systematic Reviews and Meta-Analyses
RCT: randomized controlled trial
SMD: standardized mean difference
