# Supplementary material for: Self-Administered Interventions Based on Natural Language Processing Models for Reducing Depressive and Anxiety Symptoms: Systematic Review and Meta-Analysis
Source: JMIR Ment Health. 2024 Aug 21;11:e59560. doi: 10.2196/59560 (PMC11375382; doi:10.2196/59560)
Supplement: Multimedia Appendix 2 [file mental_v11i1e59560_app2.docx]

# **Multimedia Appendix 2.** Search strategy.

**Source:** PubMed

**Date searched:** November 03, 2023

| N° | Search strategy |  |
| --- | --- | --- |
| #1 | ("Anxiety"[MeSH Terms] OR anxiety[Title/Abstract] OR "anxiety disorders"[Title/Abstract] OR "anxiety symptoms"[Title/Abstract] OR "panic attack"[Title/Abstract] OR "generalized anxiety disorder"[Title/Abstract]) | 298,199 |
| #2 | ("Depression"[MeSH Terms] OR "Depressive Disorder"[MeSH Terms] OR depression[Title/Abstract] OR "depressive symptoms"[Title/Abstract] OR "mood disorders"[Title/Abstract] OR "major depressive disorder"[Title/Abstract] OR "dysthymia"[Title/Abstract] OR "affective disorder"[Title/Abstract] or "affective disorders"[Title/Abstract]) | 539,527 |
| #3 | ("Natural Language Processing"[MeSH Terms] OR NLP[Title/Abstract] OR "text mining"[Title/Abstract] OR “Natural Language Processing”[Title/Abstract] OR “large language model”[Title/Abstract] OR LLM[Title/Abstract] OR GPT[Title/Abstract] OR “Generative Pre-trained Transformers”[Title/Abstract] OR "word embeddings"[Title/Abstract] OR "feature extraction"[Title/Abstract] OR "text classification"[Title/Abstract] OR "sentiment analysis"[Title/Abstract] OR "named entity recognition"[Title/Abstract] OR "part-of-speech tagging"[Title/Abstract] OR "text summarization"[Title/Abstract] OR "topic modeling"[Title/Abstract] OR "text segmentation"[Title/Abstract] OR “chatbot”[Title/Abstract] OR “chat-bot”[Title/Abstract] OR “conversational agent”[Title/Abstract]) | 37,585 |
| #4 | ("Non-Randomized Controlled Trials as Topic"[Mesh] OR "quasi-experimental"[Title/Abstract] OR "pre-post"[Title/Abstract] OR "non-randomized"[Title/Abstract] OR "nonrandomized"[Title/Abstract] OR "time series analysis"[Title/Abstract] OR "interrupted time series"[Title/Abstract] OR "before and after study"[Title/Abstract]) | 79,261 |
| #5 | ("Randomized Controlled Trial"[Publication Type] OR "Randomized Controlled Trials as Topic"[Mesh] OR "randomized controlled trial"[Title/Abstract] OR RCT[Title/Abstract] OR "random allocation"[Title/Abstract] OR "controlled clinical trial"[Title/Abstract] OR "randomized"[Title/Abstract] OR "placebo"[Title/Abstract]) | 1,118,758 |
| #6 | #1 OR #2 | 689,697 |
| #7 | #4 OR #5 | 1,172,607 |
| #8 | #3 AND #6 AND #7 | 59 |

**Source:** SCOPUS

**Date searched:** November 03, 2023

| N° | Search strategy |  |
| --- | --- | --- |
| #1 | (TITLE-ABS-KEY(depression) OR TITLE-ABS-KEY("depressive symptoms") OR TITLE-ABS-KEY("mood disorders") OR TITLE-ABS-KEY("major depressive disorder") OR TITLE-ABS-KEY(dysthymi*) OR TITLE-ABS-KEY("affective disorder") OR TITLE-ABS-KEY("affective disorders")) | 954,699 |
| #2 | (TITLE-ABS-KEY(anxiety) OR TITLE-ABS-KEY("anxiety disorders") OR TITLE-ABS-KEY("anxiety symptoms") OR TITLE-ABS-KEY("panic attack") OR TITLE-ABS-KEY("generalized anxiety disorder")) | 538,594 |
| #3 | (TITLE-ABS-KEY("Natural Language Processing") OR TITLE-ABS-KEY(NLP) OR TITLE-ABS-KEY("text mining") OR TITLE-ABS-KEY(“large language model”) OR TITLE-ABS-KEY(LLM) OR TITLE-ABS-KEY(GPT) OR TITLE-ABS-KEY(“Generative Pre-trained Transformers”) OR TITLE-ABS-KEY("word embeddings") OR TITLE-ABS-KEY("feature extraction") OR TITLE-ABS-KEY("text classification") OR TITLE-ABS-KEY("sentiment analysis") OR TITLE-ABS-KEY("named entity recognition") OR TITLE-ABS-KEY("part-of-speech tagging") OR TITLE-ABS-KEY("text summarization") OR TITLE-ABS-KEY("topic modeling") OR TITLE-ABS-KEY("text segmentation") OR TITLE-ABS-KEY(“chatbot”) OR TITLE-ABS-KEY(“chat-bot”) OR TITLE-ABS-KEY(“conversational agent”)) | 456,630 |
| #4 | (TITLE-ABS-KEY("quasi-experimental") OR TITLE-ABS-KEY("pre-post") OR TITLE-ABS-KEY("non-randomized") OR TITLE-ABS-KEY("nonrandomized") OR TITLE-ABS-KEY("time series analysis") OR TITLE-ABS-KEY("interrupted time series") OR TITLE-ABS-KEY("before and after study")) | 211,395 |
| #5 | (TITLE-ABS-KEY("Randomized Controlled Trial") OR TITLE-ABS-KEY(RCT) OR TITLE-ABS-KEY("random allocation") OR TITLE-ABS-KEY("controlled clinical trial") OR TITLE-ABS-KEY("randomized") OR TITLE-ABS-KEY("placebo")) | 1,794,659 |
| #6 | #1 OR #2 | 1,233,798 |
| #7 | #4 OR #5 | 1,964,818 |
| #8 | #3 AND #6 AND #7 | 141 |

**Source:** PsycINFO (via EBSCO)

**Date searched:**

| N° | Search strategy | Records |
| --- | --- | --- |
| S1 | (DE "Anxiety" OR DE "Anxiety Disorders" OR TI anxiety OR AB anxiety OR TI "anxiety disorders" OR AB "anxiety disorders" OR TI "anxiety symptoms" OR AB "anxiety symptoms" OR TI "panic attack" OR AB "panic attack" OR TI "generalized anxiety disorder" OR AB "generalized anxiety disorder") | 263,768 |
| S2 | (DE "Depression (Emotion)" OR DE "Depressive Disorders" OR TI depression OR AB depression OR TI "depressive symptoms" OR AB "depressive symptoms" OR TI "mood disorders" OR AB "mood disorders" OR TI "major depressive disorder" OR AB "major depressive disorder" OR TI "dysthymia" OR AB "dysthymia" OR TI "affective disorder" OR AB "affective disorder" OR TI "affective disorders" OR AB "affective disorders") | 337,596 |
| S3 | (TI NLP OR AB NLP OR TI "text mining" OR AB "text mining" OR TI "Natural Language Processing" OR AB "Natural Language Processing" OR TI "large language model" OR AB "large language model" OR TI LLM OR AB LLM OR TI GPT OR AB GPT OR TI "Generative Pre-trained Transformers" OR AB "Generative Pre-trained Transformers" OR TI "word embeddings" OR AB "word embeddings" OR TI "feature extraction" OR AB "feature extraction" OR TI "text classification" OR AB "text classification" OR TI "sentiment analysis" OR AB "sentiment analysis" OR TI "named entity recognition" OR AB "named entity recognition" OR TI "part-of-speech tagging" OR AB "part-of-speech tagging" OR TI "text summarization" OR AB "text summarization" OR TI "topic modeling" OR AB "topic modeling" OR TI "text segmentation" OR AB "text segmentation" OR TI “chatbot” OR AB “chatbot” OR TI “chat-bot” OR AB “chat-bot” OR TI “conversational agent” OR AB “conversational agent”) | 6,205 |
| S4 | (DE "Quasi-Experimental Studies" OR TI "quasi-experimental" OR AB "quasi-experimental" OR TI "pre-post" OR AB "pre-post" OR TI "non-randomized" OR AB "non-randomized" OR TI "nonrandomized" OR AB "nonrandomized" OR TI "time series analysis" OR AB "time series analysis" OR TI "interrupted time series" OR AB "interrupted time series" OR TI "before and after study" OR AB "before and after study") | 28,670 |
| S5 | (DE "Randomized Controlled Trials" OR TI "randomized controlled trial" OR AB "randomized controlled trial" OR TI RCT OR AB RCT OR TI "random allocation" OR AB "random allocation" OR TI "controlled clinical trial" OR AB "controlled clinical trial" OR TI "randomized" OR AB "randomized" OR TI "placebo" OR AB "placebo") | 125,726 |
| S6 | S1 OR S2 | 497,191 |
| S7 | S4 OR S5 | 150,282 |
| S8 | S3 AND S6 AND S7 | 20 |

**Source:** EMBASE (Elsevier)

**Date searched:** November 03, 2023

| N° | Search strategy | Records |
| --- | --- | --- |
| #1 | ('anxiety'/exp OR 'anxiety disorder'/exp OR 'anxiety':ti,ab OR 'anxiety disorders':ti,ab OR 'anxiety symptoms':ti,ab OR 'panic attack':ti,ab OR 'generalized anxiety disorder':ti,ab) | 671,259 |
| #2 | ('depression'/exp OR 'depressive disorder'/exp OR 'mood disorder'/exp OR 'major depressive disorder'/exp OR 'dysthymia'/exp OR 'affective disorder'/exp OR 'depression':ti,ab OR 'depressive symptoms':ti,ab OR 'mood disorders':ti,ab OR 'major depressive disorder':ti,ab OR 'dysthymia':ti,ab OR 'affective disorder':ti,ab OR 'affective disorders':ti,ab) | 987,983 |
| #3 | ('natural language processing'/exp OR 'text mining'/exp OR 'chatbot'/exp OR NLP:ti,ab OR 'Natural Language Processing':ti,ab OR 'large language model':ti,ab OR LLM:ti,ab OR GPT:ti,ab OR 'Generative Pre-trained Transformers':ti,ab OR 'word embeddings':ti,ab OR 'feature extraction':ti,ab OR 'text classification':ti,ab OR 'sentiment analysis':ti,ab OR 'named entity recognition':ti,ab OR 'part-of-speech tagging':ti,ab OR 'text summarization':ti,ab OR 'topic modeling':ti,ab OR 'text segmentation':ti,ab OR 'chatbot':ti,ab OR 'chat-bot':ti,ab OR 'conversational agent':ti,ab) | 40,004 |
| #4 | ('quasi experimental study'/exp OR 'time series analysis'/exp OR 'quasi-experimental':ti,ab OR 'pre-post':ti,ab OR 'non-randomized':ti,ab OR 'nonrandomized':ti,ab OR 'time series analysis':ti,ab OR 'interrupted time series':ti,ab OR 'before and after study':ti,ab) | 140,179 |
| #5 | ('randomized controlled trial'/exp OR 'randomized controlled trial':ti,ab OR RCT:ti,ab OR 'random allocation':ti,ab OR 'controlled clinical trial':ti,ab OR 'randomized':ti,ab OR 'placebo':ti,ab) | 1,439,642 |
| #6 | #1 OR #2 | 1,330,641 |
| #7 | #4 OR #5 | 1,542,280 |
| #8 | #3 AND #6 AND #7 | 100 |

**Source:** Web of Science

**Date searched:** November 03, 2023

| N° | Search strategy | Results |
| --- | --- | --- |
| #1 | TS=(anxiety OR "anxiety disorders" OR "anxiety symptoms" OR "panic attack" OR "generalized anxiety disorder") | 373,019 |
| #2 | TS=(depression OR "depressive symptoms" OR "mood disorders" OR "major depressive disorder" OR dysthymia OR "affective disorder" OR "affective disorders") | 673,768 |
| #3 | TS=(NLP OR "text mining" OR "Natural Language Processing" OR "large language model" OR LLM OR GPT OR "Generative Pre-trained Transformers" OR "word embeddings" OR "feature extraction" OR "text classification" OR "sentiment analysis" OR "named entity recognition" OR "part-of-speech tagging" OR "text summarization" OR "topic modeling" OR "text segmentation" OR chatbot OR "chat-bot" OR "conversational agent") | 124,906 |
| #4 | TS=("quasi-experimental" OR "pre-post" OR "non-randomized" OR "nonrandomized" OR "time series analysis" OR "interrupted time series" OR "before and after study") | 103,274 |
| #5 | TS=("randomized controlled trial" OR RCT OR "random allocation" OR "controlled clinical trial" OR randomized OR placebo) | 1,164,132 |
| #6 | #1 OR #2 | 856,471 |
| #7 | #4 OR #5 | 1,242,793 |
| #8 | #3 AND #6 AND #7 | 75 |

**Source:** IEEE Xplore

**Date searched:**

| N° | Search strategy | Results |
| --- | --- | --- |
| #1 | Full Text & Metadata:("anxiety" OR "anxiety disorders" OR "anxiety symptoms" OR "panic attack" OR "generalized anxiety disorder") | 4,347 |
| #2 | Full Text & Metadata:("depression" OR "depressive symptoms" OR "mood disorders" OR "major depressive disorder" OR "dysthymia" OR "affective disorder" OR "affective disorders") | 10,519 |
| #3 | Full Text & Metadata:(NLP OR "text mining" OR "Natural Language Processing" OR "large language model" OR LLM OR GPT OR "Generative Pre-trained Transformers" OR "word embeddings" OR "feature extraction" OR "text classification" OR "sentiment analysis" OR "named entity recognition" OR "part-of-speech tagging" OR "text summarization" OR "topic modeling" OR "text segmentation" OR "chatbot" OR "chat-bot" OR "conversational agent") | 89,600 |
| #4 | Full Text & Metadata:("quasi-experimental" OR "pre-post" OR "non-randomized" OR "nonrandomized" OR "time series analysis" OR "interrupted time series" OR "before and after study") | 11,936 |
| #5 | Full Text & Metadata:("randomized controlled trial" OR RCT OR "random allocation" OR "controlled clinical trial" OR "randomized" OR "placebo") | 28,098 |
| #6 | #1 OR #2 | 13,667 |
| #7 | #4 OR #5 | 39,235 |
| #8 | #3 AND #6 AND #7 | 277 |
