# Supplementary material for: Self-Administered Interventions Based on Natural Language Processing Models for Reducing Depressive and Anxiety Symptoms: Systematic Review and Meta-Analysis
Source: JMIR Ment Health. 2024 Aug 21;11:e59560. doi: 10.2196/59560 (PMC11375382; doi:10.2196/59560)
Supplement: Multimedia Appendix 3 [file mental_v11i1e59560_app3.docx]

**Multimedia Appendix 3.** Excluded records (n = 32).

| N° | Title | Year | Journal | Authors | Reason |
| --- | --- | --- | --- | --- | --- |
| 1 | 6.1 ARTIFICIAL INTELLIGENCE CHATBOT FOR  DEPRESSION IN YOUTH: DEVELOPMENT, USAGE, AND OUTCOMES | 2020 | Journal of the American Academy of Child and  Adolescent Psychiatry | Bunge, E. L. | Wrong publication type |
| 2 | Artificial  Intelligence-Based Chatbot for Anxiety and Depression in University Students:  Pilot Randomized Controlled Trial | 2021 | JMIR Form Res | Klos, M. C. and Escoredo, M. and Joerin, A. and  Lemos, V. N. and Rauws, M. and Bunge, E. L. | Duplicate |
| 3 | 116: Effect of  an automated conversational agent on postpartum mental health: A randomized,  controlled trial | 2020 | American Journal  of Obstetrics and Gynecology | Suharwardy, S. and Ramachandran, M. and Leonard,  S. A. and Gunaseelan, A. and Robinson, A. and Darcy, A. and Lyell, D. J. and  Judy, A. | Wrong  publication type |
| 4 | Changes in  stress, burnout, and resilience associated with an 8-week intervention with  relational agent "Woebot" | 2023 | Internet Interv | Durden, E. and Pirner, M. C. and Rapoport, S. J.  and Williams, A. and Robinson, A. and Forman-Hoffman, V. L. | Wrong outcome |
| 5 | A chatbot-based  intervention with ELME to improve stress and health-related parameters in a  stressed sample: Study protocol of a randomised controlled trial | 2023 | Frontiers in  Digital Health | Schillings, C. and Meissner, D. and Erb, B. and  Schultchen, D. and Bendig, E. and Pollatos, O. | It is a protocol |
| 6 | The Impact of a  Knowledge Discovery-Based Psychoanalytic Intervention in the Treatment of  Tuberculosis in University Students with Different Doses of Isoniazid | 2022 | Journal of  Healthcare Engineering | Xia, Z. and Tan, Y. and Yang, Y. | Retracted |
| 7 | Investigating  the Usability of Voice Assistant-Based CBT for Age-Related  Depression | 2022 | Lecture Notes in  Computer Science (including subseries Lecture Notes in Artificial  Intelligence and Lecture Notes in Bioinformatics) | Striegl, J. and Gotthardt, M. and Loitsch, C.  and Weber, G. | Wrong outcome |
| 8 | A Mental Health  Chatbot for Regulating Emotions (SERMO) - Concept and Usability Test | 2021 | IEEE  Transactions on Emerging Topics in Computing | Denecke, K. and Vaaheesan, S. and Arulnathan, A. | Wrong study  design |
| 9 | Negative Emotion  Management Using a Smart Shirt and a Robot Assistant | 2021 | IEEE Robotics  and Automation Letters | Pham, M. and Do, H. M. and Su, Z. and Bishop, A.  and Sheng, W. | Wrong study  design |
| 10 | A  Protocol-Driven, Bedside Digital Conversational Agent to Support Nurse Teams  and Mitigate Risks of Hospitalization in Older Adults: Case Control Pre-Post  Study | 2019 | J Med Internet  Res | Bott, N. and Wexler, S. and Drury, L. and  Pollak, C. and Wang, V. and Scher, K. and Narducci, S. | Wrong outcome |
| 11 | A  Quasi-Experimental Analysis of Lethal Means Assessment and Risk for  Subsequent Suicide Attempts and Deaths | 2020 | J Gen Intern Med | Boggs, J. M. and Beck, A. and Ritzwoller, D. P.  and Battaglia, C. and Anderson, H. D. and Lindrooth, R. C. | Wrong study  design |
| 12 | Smartphone-Based  Recognition of States and State Changes in Bipolar Disorder Patients | 2015 | IEEE Journal of  Biomedical and Health Informatics | Grünerbl, A. and Muaremi, A. and Osmani, V. and  Bahle, G. and Öhler, S. and Tröster, G. and Mayora, O. and Haring, C. and  Lukowicz, P. | Wrong study  design |
| 13 | Survey on  psychotherapy chatbots | 2022 | CONCURRENCY AND  COMPUTATION-PRACTICE & EXPERIENCE | Xu, B. and Zhuang, Z. Y. | Wrong  publication type |
| 14 | Usability,  Acceptability, and Effectiveness of Web-Based Conversational Agents to  Facilitate Problem Solving in Older Adults: Controlled Study | 2020 | JOURNAL OF  MEDICAL INTERNET RESEARCH | Bennion, M. R. and Hardy, G. E. and Moore, R. K.  and Kellett, S. and Millings, A. | Wrong outcome |
| 15 | Virtual Digital  Psychotherapist App-Based Treatment in Patients With Methamphetamine Use  Disorder (Echo-APP): Single-Arm Pilot Feasibility and Efficacy Study | 2023 | JMIR mHealth and  uHealth | Chen, T. and Chen, L. and Li, S. and Du, J. and  Su, H. and Jiang, H. and Wu, Q. and Zhang, L. and Bao, J. and Zhao, M. | Wrong study  design |
| 16 | Virtual human as  a new diagnostic tool, a proof of concept study in the field of major  depressive disorders | 2017 | Scientific  Reports | Philip, P. and Micoulaud-Franchi, J. A. and  Sagaspe, P. and Sevin, E. D. and Olive, J. and Bioulac, S. and Sauteraud, A. | Wrong outcome |
| 17 | Voice  Assistant-Based CBT for Depression in Students: Effects  of Empathy-Driven Dialog Management | 2022 | Lecture Notes in  Computer Science (including subseries Lecture Notes in Artificial  Intelligence and Lecture Notes in Bioinformatics) | Gotthardt, M. and Striegl, J. and Loitsch, C.  and Weber, G. | Wrong outcome |
| 18 | Effectiveness of a chatbot in improving the mental wellbeing of health workers in Malawi during the COVID-19 pandemic: A randomized, controlled trial | 2023 | medRxiv | Kleinau, E. F. and Lamba, T. and Jaskiewicz, W. and Gorentz, K. and Hungerbuehler, I. and Rahimi, D. and Kokota, D. and Maliwichi, L. and Jamu, E. S. and Zumazuma, A. and Negrão, M. and Mota, R. and Khouri, Y. and Kapps, M. | Pre-print |
| 19 | A Multilingual Digital Mental Health and Well-Being Chatbot (ChatPal): Pre-Post Multicenter Intervention Study | 2023 | JOURNAL OF MEDICAL INTERNET RESEARCH | Potts, C. and Lindstroem, F. and Bond, R. and Mulvenna, M. and Booth, F. and Ennis, E. and Parding, K. and Kostenius, C. and Broderick, T. and Boyd, K. and Vartiainen, A. K. and Nieminen, H. and Burns, C. and Bickerdike, A. and Kuosmanen, L. and Dhanapala, I. and Vakaloudis, A. and Cahill, B. and MacInnes, M. and Malcolm, M. and O'Neill, S. | Wrong outcome |
| 20 | Understanding Digital Mental Health Needs and Usage with an Artificial Intelligence–Led Mental Health App (Wysa) during the COVID-19 Pandemic: Retrospective Analysis | 2023 | JMIR Formative Research | Sinha, C. and Meheli, S. and Kadaba, M. | Wrong intervention |
| 21 | Effects of artificial intelligence on English speaking anxiety and speaking performance: A case study | 2021 | Expert Systems | El Shazly, R. | Wrong outcome |
| 22 | Pilot randomised controlled trial of Help4Mood, an embodied virtual agent-based system to support treatment of depression | 2016 | J Telemed Telecare | Burton C, Szentagotai Tatar A, McKinstry B, Matheson C, Matu S, Moldovan R, Macnab M, Farrow E, David D, Pagliari C, Serrano Blanco A, Wolters M | Intervention was not self-applied |
| 23 | Electronic problem-solving treatment: description and pilot study of an interactive media treatment for depression | 2012 | JMIR Res Protoc | Cartreine JA, Locke SE, Buckey JC, Sandoval L, Hegel MT. | Wrong  intervention |
| 24 | AVATAR therapy for auditory verbal hallucinations in people with psychosis: a single-blind, randomised controlled trial | 2018 | Lancet Psychiatry | Craig TK, Rus-Calafell M, Ward T, Leff JP, Huckvale M, Howarth E, Emsley R, Garety PA. | Intervention was not self-applied |
| 25 | One-year randomized trial comparing virtual reality-assisted therapy to cognitive-behavioral therapy for patients with treatment-resistant schizophrenia | 2021 | NPJ Schizophr | Dellazizzo L, Potvin S, Phraxayavong K, Dumais A. | Intervention was not self-applied |
| 26 | Effectiveness of self-guided app-based virtual reality cognitive behavior therapy for acrophobia: a randomized clinical trial | 2019 | JAMA Psychiatry | Donker T, Cornelisz I, van Klaveren C, van Straten A, Carlbring P, Cuijpers P, van Gelder JL. | Wrong intervention |
| 27 | Virtual reality therapy for refractory auditory verbal hallucinations in schizophrenia: a pilot clinical trial | 2018 | Schizophr Res | du Sert OP, Potvin S, Lipp O, Dellazizzo L, Laurelli M, Breton R, Lalonde P, Phraxayavong K, O'Connor K, Pelletier JF, Boukhalfi T, Renaud P, Dumais A. | Wrong  intervention |
| 28 | Computer-assisted avatar-based treatment for dysfunctional beliefs in depressive inpatients: a pilot study | 2021 | Front Psychiatry | Kocur M, Dechant M, Wolff C, Nothdurfter C, Wetter TC, Rupprecht R, Shiban Y. | Wrong intervention |
| 29 | Therapist-led and self-led one-session virtual reality exposure therapy for public speaking anxiety with consumer hardware and software: a randomized controlled trial | 2019 | J Anxiety Disord | Lindner P, Miloff A, Fagernäs S, Andersen J, Sigeman M, Andersson G, Furmark T, Carlbring P. | Wrong  intervention |
| 30 | Automated virtual reality exposure therapy for spider phobia vs. in-vivo one-session treatment: a randomized non-inferiority trial | 2019 | Behav Res Ther | Miloff A, Lindner P, Dafgård P, Deak S, Garke M, Hamilton W, Heinsoo J, Kristoffersson G, Rafi J, Sindemark K, Sjölund J, Zenger M, Reuterskiöld L, Andersson G, Carlbring P. | Wrong intervention |
| 31 | Avatar-based depression self-management technology: promising approach to improve depressive symptoms among young adults | 2013 | Appl Nurs Res | Pinto MD, Hickman Jr RL, Clochesy J, Buchner M. | Wrong  intervention |
| 32 | Randomized controlled trial of a computerized interactive media-based problem solving treatment for depression | 2021 | Behav Ther | Sandoval LR, Buckey JC, Ainslie R, Tombari M, Stone W, Hegel MT. | Wrong intervention |
