# Supplementary material for: Self-Administered Interventions Based on Natural Language Processing Models for Reducing Depressive and Anxiety Symptoms: Systematic Review and Meta-Analysis
Source: JMIR Ment Health. 2024 Aug 21;11:e59560. doi: 10.2196/59560 (PMC11375382; doi:10.2196/59560)
Supplement: Multimedia Appendix 4 [file mental_v11i1e59560_app4.docx]

**Multimedia Appendix 4**. Included records (n = 21).

| N° | Title | Authors | Journal | Year | Source |
| --- | --- | --- | --- | --- | --- |
| 1 | Artificial Emotional Intelligence in Socially Assistive Robots for Older Adults: A Pilot Study | Abdollahi, H. and Mahoor, M. H. and Zandie, R. and Siewierski, J. and Qualls, S. H. | IEEE Transactions on Affective Computing | 2023 | https://ieeexplore.ieee.org/abstract/document/9684685 |
| 2 | A chatbot for mental health support: exploring the impact of Emohaa on reducing mental distress in China | Sabour, S. and Zhang, W. and Xiao, X. Y. and Zhang, Y. W. and Zheng, Y. H. and Wen, J. X. and Zhao, J. L. and Huang, M. L. | FRONTIERS IN DIGITAL HEALTH | 2023 | https://www.ncbi.nlm.nih.gov/pmc/articles/PMC10193040/ |
| 3 | Feasibility and impact of a mental health chatbot on postpartum mental health: a randomized controlled trial | Suharwardy, S. and Ramachandran, M. and Leonard, S. A. and Gunaseelan, A. and Lyell, D. J. and Darcy, A. and Robinson, A. and Judy, A. | AJOG Glob Rep | 2023 | https://pubmed.ncbi.nlm.nih.gov/37560011/ |
| 4 | Assessing the Impact of Conversational Artificial Intelligence in the Treatment of Stress and Anxiety in Aging Adults: Randomized Controlled Trial | Danieli, M. and Ciulli, T. and Mousavi, S. M. and Silvestri, G. and Barbato, S. and Di Natale, L. and Riccardi, G. | JMIR Mental Health | 2022 | https://pubmed.ncbi.nlm.nih.gov/36149730/ |
| 5 | Effectiveness of a chatbot for eating disorders prevention: A randomized clinical trial | Fitzsimmons‐Craft, Ellen E. and Chan, William W. and Smith, Arielle C. and Firebaugh, Marie‐Laure and Fowler, Lauren A. and Topooco, Naira and DePietro, Bianca and Wilfley, Denise E. and Taylor, C. Barr and Jacobson, Nicholas C. | International Journal of Eating Disorders | 2022 | https://pubmed.ncbi.nlm.nih.gov/35274362/ |
| 6 | Chatbot-Delivered Cognitive Behavioral Therapy in Adolescents With Depression and Anxiety During the COVID-19 Pandemic: Feasibility and Acceptability Study | Nicol, G. and Wang, R. Y. and Graham, S. and Dodd, S. and Garbutt, J. | JMIR Formative Research | 2022 | https://pubmed.ncbi.nlm.nih.gov/36413390/ |
| 7 | Mental Health Chatbot for Young Adults With Depressive Symptoms During the COVID-19 Pandemic: Single-Blind, Three-Arm Randomized Controlled Trial | He, Y. H. and Yang, L. and Zhu, X. K. and Wu, B. and Zhang, S. and Qian, C. L. and Tian, T. | JOURNAL OF MEDICAL INTERNET RESEARCH | 2022 | https://pubmed.ncbi.nlm.nih.gov/36355633/ |
| 8 | Using AI chatbots to provide self-help depression interventions for university students: A randomized trial of effectiveness | Liu, H. and Peng, H. M. and Song, X. Y. and Xu, C. Z. and Zhang, M. | INTERNET INTERVENTIONS | 2022 | https://pubmed.ncbi.nlm.nih.gov/35059305/ |
| 9 | A Virtual Coach (Motibot) for Supporting Healthy Coping Strategies Among Adults With Diabetes: Proof-of-Concept Study | Bassi, G. and Giuliano, C. and Perinelli, A. and Forti, S. and Gabrielli, S. and Salcuni, S. | JMIR Human Factors | 2022 | https://pubmed.ncbi.nlm.nih.gov/35060918/ |
| 10 | Efficacy of Zemedy, a Mobile Digital Therapeutic for the Self-management of Irritable Bowel Syndrome: Crossover Randomized Controlled Trial | Hunt, M. and Miguez, S. and Dukas, B. and Onwude, O. and White, S. | JMIR Mhealth Uhealth | 2021 | https://pubmed.ncbi.nlm.nih.gov/33872182/ |
| 11 | Artificial intelligence⇓based chatbot for anxiety and depression in university students: Pilot randomized controlled trial | Klos, M. C. and Escoredo, M. and Joerin, A. and Lemos, V. N. and Rauws, M. and Bunge, E. L. | JMIR Formative Research | 2021 | https://formative.jmir.org/2021/8/e20678 |
| 12 | A Therapeutic Relational Agent for Reducing Problematic Substance Use (Woebot): Development and Usability Study | Prochaska, J. J. and Vogel, E. A. and Chieng, A. and Kendra, M. and Baiocchi, M. and Pajarito, S. and Robinson, A. | J Med Internet Res | 2021 | https://www.jmir.org/2021/3/e24850/ |
| 13 | Efficacy of mobile app-based interactive cognitive behavioral therapy using a chatbot for panic disorder | Oh, J. and Jang, S. and Kim, H. and Kim, J. J. | International Journal of Medical Informatics | 2020 | https://pubmed.ncbi.nlm.nih.gov/32446158/ |
| 14 | Use of the Chatbot "Vivibot" to Deliver Positive Psychology Skills and Promote Well-Being Among Young People After Cancer Treatment: Randomized Controlled Feasibility Trial | Greer, S. and Ramo, D. and Chang, Y. J. and Fu, M. and Moskowitz, J. and Haritatos, J. | JMIR Mhealth Uhealth | 2019 | https://mhealth.jmir.org/2019/10/e15018/authors |
| 15 | Using Psychological Artificial Intelligence (Tess) to Relieve Symptoms of Depression and Anxiety: Randomized Controlled Trial | Fulmer, R. and Joerin, A. and Gentile, B. and Lakerink, L. and Rauws, M. | JMIR Mental Health | 2018 | https://pubmed.ncbi.nlm.nih.gov/30545815/ |
| 16 | Delivering Cognitive Behavior Therapy to Young Adults With Symptoms of Depression and Anxiety Using a Fully Automated Conversational Agent (Woebot): A Randomized Controlled Trial | Fitzpatrick, K. K. and Darcy, A. and Vierhile, M. | JMIR Mental Health | 2017 | https://mental.jmir.org/2017/2/e19 |
| 17 | Manage your life online: a web-based randomized controlled trial evaluating the effectiveness of a problem-solving intervention in a student sample | Bird T, Mansell W, Wright J, Gaffney H, Tai S. | Behav Cogn Psychother. | 2018 | https://pubmed.ncbi.nlm.nih.gov/29366432/ |
| 18 | Manage your life online (MYLO): a pilot trial of a conversational computer-based intervention for problem solving in a student sample | Gaffney H, Mansell W, Edwards R, Wright J. | Behav Cogn Psychother | 2014 | https://pubmed.ncbi.nlm.nih.gov/23899405/ |
| 19 | Mobile app-based chatbot to deliver cognitive behavioral therapy and psychoeducation for adults with attention deficit: A development and feasibility/usability study | Jang S, Kim JJ, Kim SJ, Hong J, Kim S, Kim E | Int J Med Inform | 2023 | https://pubmed.ncbi.nlm.nih.gov/33799055/ |
| 20 | Promoting fertility awareness and preconception health using a chatbot: a randomized controlled trial | Maeda E, Miyata A, Boivin J, Nomura K, Kumazawa Y, Shirasawa H, Saito H, Terada Y. | Reprod Biomed Online | 2020 | https://pubmed.ncbi.nlm.nih.gov/33039321/ |
| 21 | A randomized controlled trial of a therapeutic relational agent for reducing substance misuse during the COVID-19 pandemic | Prochaska JJ, Vogel EA, Chieng A, Baiocchi M, Maglalang DD, Pajarito S, Weingardt KR, Darcy A, Robinson A. | Drug Alcohol Depend | 2021 | https://pubmed.ncbi.nlm.nih.gov/34507061/ |
