# Supplementary material for: Self-Administered Interventions Based on Natural Language Processing Models for Reducing Depressive and Anxiety Symptoms: Systematic Review and Meta-Analysis
Source: JMIR Ment Health. 2024 Aug 21;11:e59560. doi: 10.2196/59560 (PMC11375382; doi:10.2196/59560)

**Multimedia Appendix 6.** Risk of bias for individual studies for the outcome of depressive symptoms.


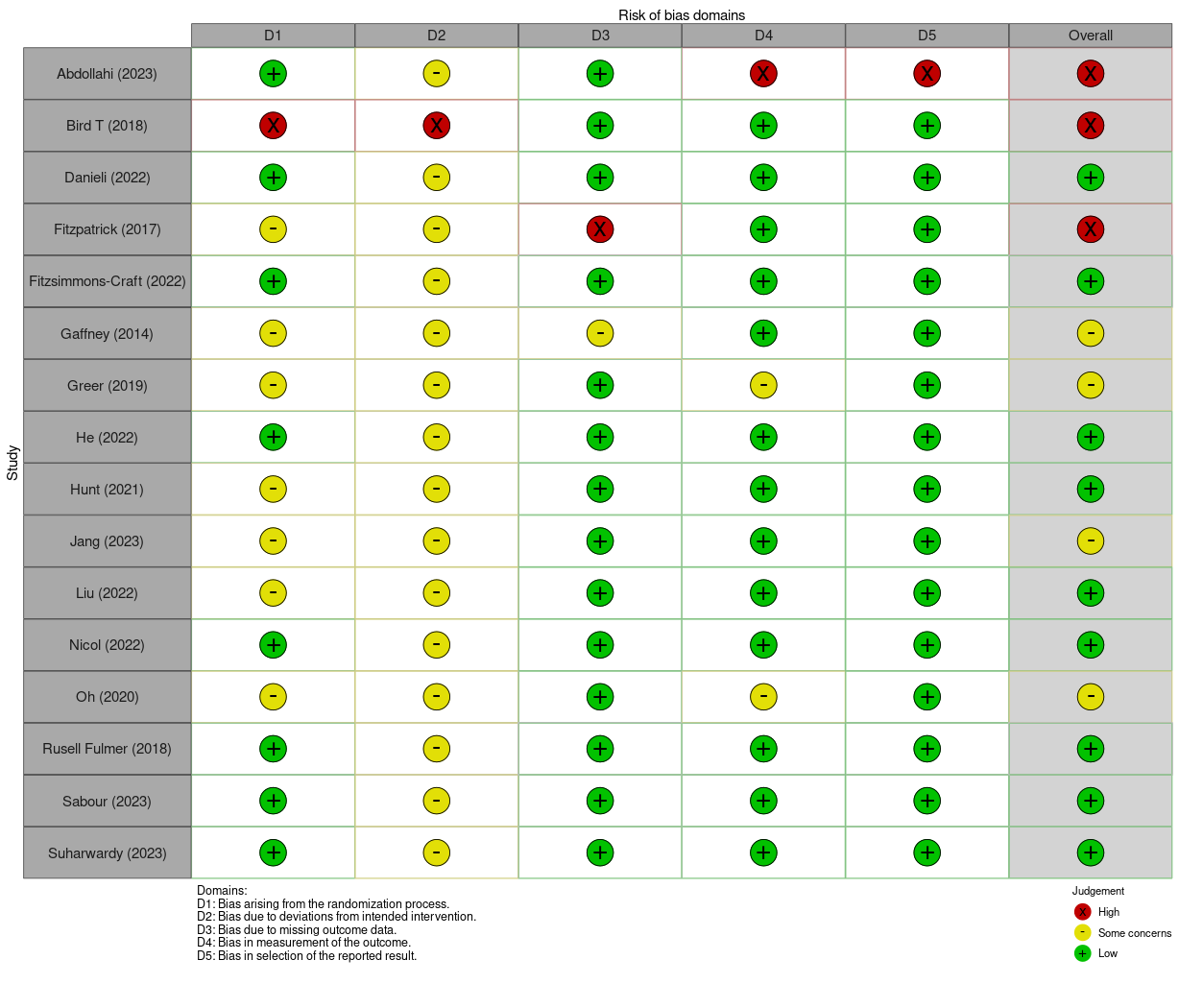

Supplement: Multimedia Appendix 6 [file mental_v11i1e59560_app6.docx]
